# Effects of a single-dose denosumab on glucose and lipid homeostasis in young infertile men

**DOI:** 10.1007/s12020-026-04683-8

**Published:** 2026-06-25

**Authors:** Emil Brink Wriedt, Mads Joon Jorsal, Sam Kafai Yahyavi, Li Juel Mortensen, Ebbe Eldrup, Anders Juul, Rune Holt, Martin Blomberg Jensen

**Affiliations:** 1 Division of Translational Endocrinology, Department of Endocrinology and Internal Medicine, Copenhagen University Hospital - Herlev and Gentofte, Herlev, Denmark; 2 Group of Skeletal, Mineral and Gonadal Endocrinology, Department of Growth and Reproduction, Copenhagen University Hospital – Rigshospitalet, Copenhagen, Denmark; 3 Department of Growth and Reproduction, Copenhagen University Hospital - Rigshospitalet, Copenhagen, Denmark; 4 Department of Clinical Medicine, Copenhagen University Hospital, Copenhagen, Denmark; 5 International Centre for Research and Research Training in Endocrine Disruption of Male Reproduction and Child Health (EDMaRC), Copenhagen University Hospital – Rigshospitalet, Copenhagen, Denmark

**Keywords:** Denosumab, Infertile, Men, Glucose, HbA1c, Cortisol

## Abstract

**Purpose:**

Infertile men have an increased prevalence of metabolic syndrome. Denosumab has been shown to lower HbA1c in older patients with impaired glucose tolerance. We hypothesized that denosumab will lower HbA1c in a cohort of young infertile men.

**Methods:**

This was a secondary analysis of a single-center, placebo-controlled randomized clinical trial comprising infertile men with no serious co-morbidities. Men were randomized 1:1 to receive a single s.c. injection of 60 mg denosumab or placebo. Prespecified secondary outcome variables included HbA1c, fasting glucose, fasting insulin, C-peptide, while the primary end point semen quality has previously been reported.

**Results:**

We randomized 100 infertile men and 92 were included in the final analysis. There were no differences between denosumab and placebo groups at day 80 or 160 for HbA1c, LDL, HDL or total cholesterol. For fasting patients (*n* = 44) there were no differences in plasma glucose, plasma insulin, C-peptide, or HOMA-IR. At day 160 alanine aminotransferase was higher in the denosumab group compared to placebo 8.3 U/L (95% CI 3.3 to 13.3). In a subgroup with high baseline serum cortisol, denosumab reduced HbA1c by 2.5 mmol/mol (95% CI -5.0 to -0.1) compared to placebo.

**Conclusion:**

A single dose of denosumab had no effect on glucose- or lipid homeostasis but induced a small increase in serum ALT in young infertile men with no serious co-morbidities.

**Clinical trial registration number:**

NCT03030196.

**Supplementary Information:**

The online version contains supplementary material available at 10.1007/s12020-026-04683-8.

## Introduction

Impaired gonadal function is linked with poor skeletal health [1]. Especially hypogonadism is associated with altered bone mineral content, and some studies have shown that infertile men with low testosterone also may have increased osteoclast activity, reduced bone mass, and increased risk of osteoporosis [1]. Infertile men also have a higher prevalence of metabolic syndrome, obesity, and an increased risk of type 2 diabetes (T2DM) [2, 3]. This may in part be due to hypogonadism, but the exact etiology remains to be clarified. Recently, serum levels of the bone factor RANKL have been associated with glucose and lipid metabolism in infertile men [4]. Moreover, FSH stimulation of human-derived adipocyte cell lines increased the release of RANKL in vitro, which highlights that RANKL possibly may mediate some of the systemic effects in men with impaired gonadal function [4].

RANKL activates the receptor RANK that induces osteoclastogenesis until the RANKL-RANK interaction is blocked by OPG, a decoy receptor that inhibits RANKL signaling and thereby bone resorption [5]. This knowledge prompted the development of denosumab, a human monoclonal IgG2 antibody that specifically targets and inhibits RANKL, now recognized as a highly effective treatment for osteoporosis [6]. Several studies have suggested effects of the RANKL signaling pathway outside the skeleton, particularly in the immune system, skeletal muscle, and more recently, in male reproduction [7–11]. The RANKL/RANK/OPG signaling axis has also emerged as a potential target for modulating glucose metabolism and insulin sensitivity [12–18]. Soluble RANKL (sRANKL) has even been identified as a potential prognostic marker for the development of T2DM [18]. Additionally, RANKL inhibition has been linked to increased beta-cell proliferation and mass in pre-clinical models of both type 1 and type 2 diabetes [13, 16]. Epidemiological studies have shown a reduced incidence of T2DM in osteoporotic patients treated with denosumab [19–21]. However, the direct influence of denosumab on glucose metabolism in humans has not been characterized. Clinical trials evaluating denosumab’s effects on glucose parameters have yielded conflicting results [22–29]. A recent meta-analysis reported improvements in fasting plasma glucose (FPG) and HbA1c after denosumab treatment among subjects with impaired glucose tolerance (IGT), but no significant effects were observed in subjects with normal glucose tolerance (NGT) [30]. As most of these studies were conducted in older osteoporotic women, our cohort of young infertile men – a group rarely treated with denosumab – could provide novel insights into the relationship between denosumab and glucose homeostasis.

This study is a secondary analysis of a randomized placebo-controlled clinical trial, designed to investigate the effect of denosumab on semen quality in infertile men. We hypothesized that inhibition of RANKL with denosumab influences glucose and lipid homeostasis in infertile men, as this patient population is linked with poorer metabolic health.

## Materials and methods

### Study design and participants

Copenhagen First (NCT03030196) was a single-center, double-blinded, parallel-group, randomized clinical trial conducted between 2017 and 2019 at the Department of Growth and Reproduction, Rigshospitalet, Denmark. The detailed study design has been described previously [31]. Informed written consent was obtained from all participants. All men eligible for inclusion were part of an infertile couple whose semen analysis had shown impaired semen quality, no serious co-morbidities, and who had been referred to the Department of Growth and Reproduction, Rigshospitalet, Denmark [31]. All included men were randomly assigned (1:1) to a single s.c. injection with denosumab (60 mg/mL, Prolia, Amgen, US) or s.c. injection with 1 mL saline (0.9%). To prevent denosumab-induced hypocalcemia, all participants were recommended daily cholecalciferol (25 µg) and calcium carbonate (400 mg) (Actavis, Denmark) supplementation for the 180-day experimental period.

### Study outcomes

The primary endpoint of the study was change in sperm production (total motile, progressively motile sperm, sperm count, sperm concentration), and has been reported previously along with serum testosterone [31].

This study was a secondary analysis of the Copenhagen First Trial. Change in HbA1c, fasting glucose, fasting insulin, C-peptide, and lipid-profile were pre-specified as secondary outcomes.

### Biochemical analysis

All blood samples were drawn between 08:00 and 10:00 in the morning. All participants were instructed to do an overnight fast. Cortisol, sRANKL, and OPG were measured from frozen serum after trial completion at the Department of Growth and Reproduction, Copenhagen University Hospital, Rigshospitalet. sRANKL (#BI-20462, Biomedica, Austria) and OPG (#BI-20403, Biomedica, Austria) were analysed using commercial ELISA kits, validated in previous studies [7, 32]. Cortisol and testosterone were measured using an isotope-dilution TurboFlow-LC-MS/MS method [33]. The following analytes were measured at the Department of Clinical Biochemistry, Copenhagen University Hospital, Rigshospitalet with accredited analyses: HbA1c was measured on a Tosoh G8 with a coefficient of variation (CV) of 2.8%. Alkaline phosphatase (CV 5.5%), blood glucose (CV 4%), insulin (CV 5%), C-peptide (CV 6%), total cholesterol (CV 5%), low-density lipoprotein (LDL; CV 4%), high-density lipoprotein (HDL; CV 4%), and triglycerides (CV 5%) were all measured on a Cobas 8000 (Roche). Alanine aminotransferase (CV 5%) was measured on a Cobas Pro (Roche).

Lean and fat mass were determined by dual-energy X-ray absorption (DXA) scans performed on Hologic CDR 1000/W densitometer (Hologic, Inc., Bedford, MA, USA).

Indices of insulin resistance were calculated using the formula:

HOMA-IR = (Fasting insulin µU/mL * Fasting glucose mmol/L) / 22.5.

A conversion factor of 6 was used to convert Insulin from pmol/L to µU/mL.

### Statistical Analysis

Descriptive statistics are presented in Tables 1 and 2. Continuous variables are reported as means with standard deviation (SD), and categorical variables are presented as frequencies and percentages. Sampling distribution of variables was assessed with density- and histogram plots. Unadjusted between-group differences between outcomes at day 80 and 160 were obtained using t-tests for Gaussian distributed values and Mann-Whitney U tests for skewed distributions. Adjusted differences for baseline values were estimated using a linear mixed effects model [34]. Time and treatment were coded as fixed effects with subject identity coded as a random intercept. Model metrics were evaluated by graphical methods. Confidence intervals for skewed outcomes were obtained by estimating the mean value of 10,000 bootstrap resamples using the ‘boot’ package in R. A cluster-based bootstrap on subject level was applied for the mixed model to preserve correct within-subject correlation using the ‘lmersampler’ package in R. Resampling distribution was assessed for normality using a histogram. Subgroup analyses were undertaken for baseline values of age, BMI, lipids, sRANKL, OPG, HOMA-IR, DXA-derived body mass measures, testosterone, and ALT to evaluate for heterogeneity of treatment effect. Further potential subgroups were identified using the ‘SIDES’ package in R [35], a machine learning algorithm specifically designed for exploratory subgroup identification in RCTs. All subgroups were evaluated for significance using a test for interaction. No correction for multiple testing was done due to the exploratory nature of this study. Contrasts for the subgroup analysis were calculated using estimated marginal means.

**Table 1:** Baseline characteristics. Data presented as means and SD or frequencies and percentages

Baseline Table (mean (SD))	Denosumab	Placebo
Included	48	49
Age	33.6 (6)	33.8 (6)
Height (cm)	183.6 (8.6)	183.9 (6.3)
Weight (kg)	89.6 (12.8)	87.85 (14.2)
BMI (kg/m^2^)	26.6 (3.8)	26.0 (3.6)
Smokers		
- Active	4 (8.3%)	6 (12.2%)
- Never	27 (56.2%)	31 (63.3%)
- Previous	17 (35.4%)	12 (24.5%)
Glucose (mmol/L)	5.2 (0.9)	4.96 (0.55)
Insulin (pmol/L)	109.0 (192.6)	80.8 (79.2)
C-peptide (pmol/L)	832.9 (662.6)	752.0 (379)
HbA1c (mmol/mol)	32.7 (3.1)	32.4 (3.4)
HOMA-IR	5.2 (12.13)	3.1 (3.4)
Total Cholesterol (mmol/L)	4.9 (0.9)	4.7 (0.9)
HDL (mmol/L)	1.4 (0.3)	1.4 (0.3)
LDL (mmol/L)	3.1 (0.9)	3.1 (0.8)
Triglycerides (mmol/L)	1.4 (1.1)	1.0 (0.6)
ALT (U/L)	32.3 (16.6)	32.2 (17.4)
Cortisol (nmol/L)	306 (95)	326 (102)
Testosterone (nmol/L)	17.4 (6.4)	19.3 (8)
Hypogonadism (%)	7 (14.6)	5 (10.4)
Total Fat Mass (kg)	24.5 (8.9)	23.5 (8.2)
Android Fat Mass (kg)	2.3 (1.2)	2.2 (1.2)
Gyneoid Fat Mass (kg)	4.0 (1.5)	3.7 (1.3)
Total Lean Mass (kg)	60.9 (6.5)	60.3 (8.0)
Android Lean Mass (kg)	4.1 (0.5)	4.1 (0.6)
Gyneoid Lean Mass (kg)	9.8 (1.2)	9.7 (1.4)

Abbreviations: ALT, alanine aminotransferase; BMI, body mass index; HbA1c, glycated hemoglobin A1c; HOMA-IR, homeostatic model assessment of insulin resistance; LDL, low-density lipoprotein; HDL, high-density lipoprotein. Hypogonadism defined by serum testosterone < 10.4 nmol/L

**Table 2:** Outcomes at day 80 and 160

Outcome	Denosumab (*n* = 46)		Placebo (*n* = 46)		Unadjusted difference		Adjusted difference^a^	
	Day 80 (mean (SD))	Day 160 (mean (SD))	Day 80 (mean (SD))	Day 160 (mean (SD))	Day 80 (95% CI)	Day 160 (95% CI)	Day 80 (95% CI)	Day 160 (95% CI)
Glucose (mmol/L)^b^	5.0 (0.5)	5.1 (0.4)	5.3 (0.5)	5.2 (0.5)	-0.2 (-0.5 to 0.06)	-0.2 (-0.5 to 0.1)	0.1 (-0.1 to 0.3)	0.2 (-0.1 to 0.4)
Insulin (pmol/L)^b, d^	74.8 (50.5)	70.2 (36.9)	97.7 (71.3)	61.3 (55.0)	-23 (-63 to 9)	-9 (-51 to 11)	-1.1 (-27 to 24)	7.1 (-27 to 34.6)
C-peptide (pmol/L)^b^	707.2 (304)	676.9 (219)	830.6 (359)	780.6 (301)	-123 (-326 to 79)	-73 (-258 to 111)	-29 (-170 to 112)	-9 (-150 to 132)
HOMA-IR^b, d^	2.9 (2.2)	2.7 (1.56)	4.0 (3.5)	3.4 (2.9)	-1.1 (-3.1 to 0.3)	-0.7 (-2.6 to 0.4)	0.04 (-1.2 to 1.2)	0.05 (-1.3 to 1.8)
HbA1c (mmol/mol)	32.7 (3.1)	32.7 (2.9)	32.5 (3.0)	32.8 (3.5)	0.3 (-1-0 to 1.5)	-0.07 (-1.4 to 1.3)	0.05 (-0.6 to 0.7)	-0.3 (-0.9 to 0.3)
HDL (mmol/L)	1.4 (0.3)	1.36 (0.3)	1.3 (0.3)	1.31 (0.3)	0.03 (-0.08 to 0.2)	0.06 (-0.06 to 0.17)	0.02 (-0.2 to 0.2)	0.1 (-0.1 to 0.3)
LDL (mmol/L)	3.2 (0.9)	3.2 (0.9)	3.1 (1.0)	3.1 (0.9)	0.07 (-0.3 to 0.5)	0.2 (-0.2 to 0.5)	0.03 (-0.04 to 0.1)	0.05 (-0.01 to 0.12)
Total Cholesterol (mmol/L)	4.8 (0.9)	4.9 (0.9)	4.7 (1.0)	4.6 (0.9)	0.1 (-0.3 to 0.5)	0.3 (-0.09 to 0.7)	-0.1 (-0.3 to 0.2)	0.1 (-0.1 to 0.3)
Triglycerides (mmol/L)^d^	1.2 (0.6)	1.4 (1.0)	1.1 (0.6)	1.1 (0.4)	0.09 (-0.1 to 0.3)	**0.3 (0.1 to 0.8)** ^**c**^	-0.2 (-0.4 to 0.01)	0.06 (-0.2 to 0.3)
ALT (U/L)^d^	35.3 (19.8)	38.1 (20.0)	29.2 (15.5)	28.5 (14.1)	6.1 (-0.8 to 13.6)	**9.5 (2.9 to 16.9)** ^**^	4.9 (-0.4 to 10.3)	8.3 (3.3 to 13.3)**
Cortisol (nmol/L)	354.6 (107.8)	342.7 (122.5)	395.7 (106.6)	371.9 (95.3)	-41 (-85.5 to 3.3)	-29 (-75 to 17)	-22 (-66 to 21)	-9 (-53 to 34)
Total Fat Mass (kg)	-	25.1 (9.1)	-	23.5 (8.2)	-	1.6 (-2.0 to 5.2)	-	0.6 (-0.2 to 1.4)
Android Fat Mass (kg)	-	2.4 (1.3)	-	2.2 (1.2)	-	0.2 (-0.3 to 0.7)	-	0.1 (-0.01 to 0.2)
Gyneoid Fat Mass (kg)	-	4.1 (1.5)	-	3.8 (1.2)	-	0.3 (-0.3 to 0.9)	-	0.07 (-0.08 to 0.2)
Total Lean Mass (kg)	-	60.8 (6.5)	-	60.3 (8.4)	-	0.5 (-2.6 to 3.6)	-	-0.1 (-0.7 to 0.4)
Android Lean Mass (kg)	-	4.1 (5.3)	-	4.2 (0.6)	-	-0.08 (-0.3 to 0.2)	-	-0.01 (-0.09 to 0.07)
Gyneoid Lean Mass (kg)	-	9.8 (1.2)	-	9.6 (1.5)	-	0.2 (-0.3 to 0.7)	-	0.05 (-0.09 to 0.2)

Estimates not in bold are non-significant. ^a^Estimated with a linear mixed effects model to adjust for baseline levels. ^b^Only fasting patients (*n* = 22 in each group). ^c^Significant as evaluated by confidence intervals, but non-significant by Mann-Whitney-U test. ^d^Confidence Intervals calculated using a non-parametric bootstrap resampling for unadjusted differences and a cluster-based bootstrap resampling for adjusted differences to preserve within-subject correlation. *P-value < 0.05, **P-value < 0.01. Abbreviations: ALT, alanine aminotransferase; BMI, body mass index; HbA1c, glycated hemoglobin A1c; HOMA-IR, homeostatic model assessment of insulin resistance; LDL, low-density lipoprotein; HDL, high-density lipoprotein

Data was analyzed based on an intention-to-treat principle, except for glucose, insulin, C-peptide, and HOMA-IR which were analyzed in the subset of patients who were fasting. Results are presented as means with 95% confidence intervals unless otherwise stated. Power calculations were based on the primary end point, semen quality [31].

All statistical analyses were performed in R statistical software version 4.4.1. (R Core Team 2021. R: A Language and Environment for Statistical Computing. R Foundation for Statistical Computing, Vienna, Austria. (http://www.R-project.org). A p-value of < 0.05 was considered statistically significant.

## Results

### Flow and Baseline characteristics

**Fig. 1:**
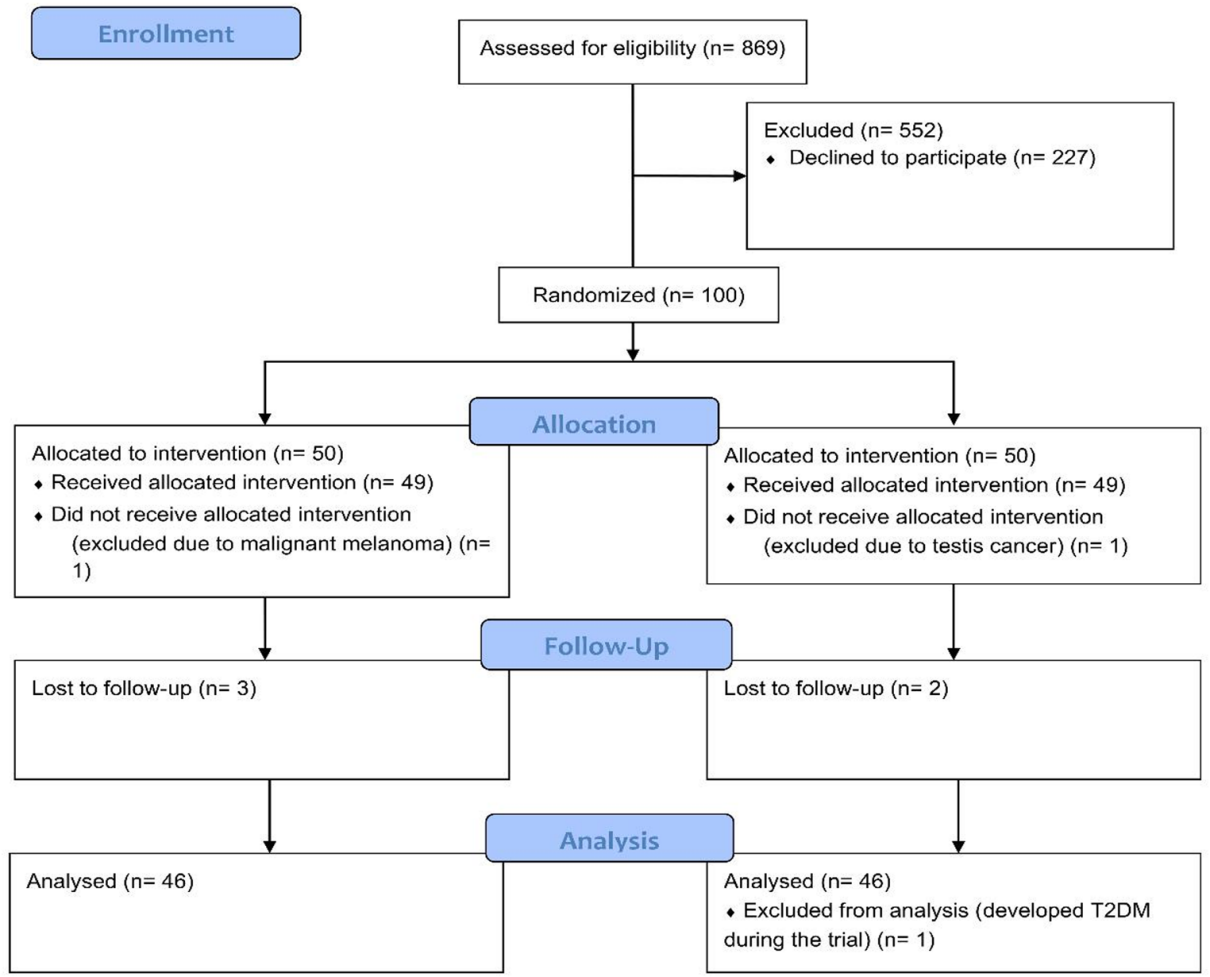
Flowchart – from enrollment to follow-up

Patient flow is shown in Fig. 1.

In short, 869 men were assessed for eligibility. Of these, 100 men were included and randomized to receive either denosumab or placebo. 2 patients were excluded due to malignant melanoma and testicular cancer, respectively. 93 men completed the study. In this secondary analysis, with focus on glucose metabolism, we excluded 1 additional patient from the analysis since he had a HbA1c of 76 mmol/mol at baseline and was diagnosed with T2DM shortly after the trial ended. One patient in the placebo group had an HbA1c of 42 mmol/mol and was therefore classified as having prediabetes. Only 44 patients delivered fasting blood samples at baseline, day 80 and 160 as instructed (*n* = 22 and *n* = 22 in the denosumab and placebo group, respectively). Baseline characteristics for fasting patients are shown in Supplementary Table 1. 12 patients had a testosterone level below 10.4 nmol/L and were assigned a biochemical diagnosis of hypogonadism. All hypogonadal patients were classified as having primary hypogonadism although some of them may have functional hypogonadism since LH was within the normal range; no cases of hypogonadotropic hypogonadism or obstructive azoospermia were included.

Baseline values were comparable between both groups except for glucose, insulin, C-peptide and HOMA-IR due to most patients not fasting as instructed (Table 1). The mean age was 33.7 years (SD 6.0) and BMI was 26.3 kg/m^2^ (SD 3.7). The mean HbA1c was 32.6 mmol/mol (SD 3.2) and total cholesterol was 4.8 mmol/L (SD 1.0).

### Glucose, lipids, ALT and anthropometrics

There were no differences in HbA1c between groups in the unadjusted nor adjusted analyses at day 80 and day 160 (Table 2).

ALT was higher in the denosumab group compared to placebo at day 160 with a mean difference of 8.3 U/L (3.3 to 13.3) (*p* = 0.015) (Table 2). This effect was 1.2 U/L higher in the unadjusted analysis. The numerical increase was of a similar magnitude at day 80 (Table 2), although this difference did not reach statistical significance (*p* = 0.14).

**Fig. 2:**
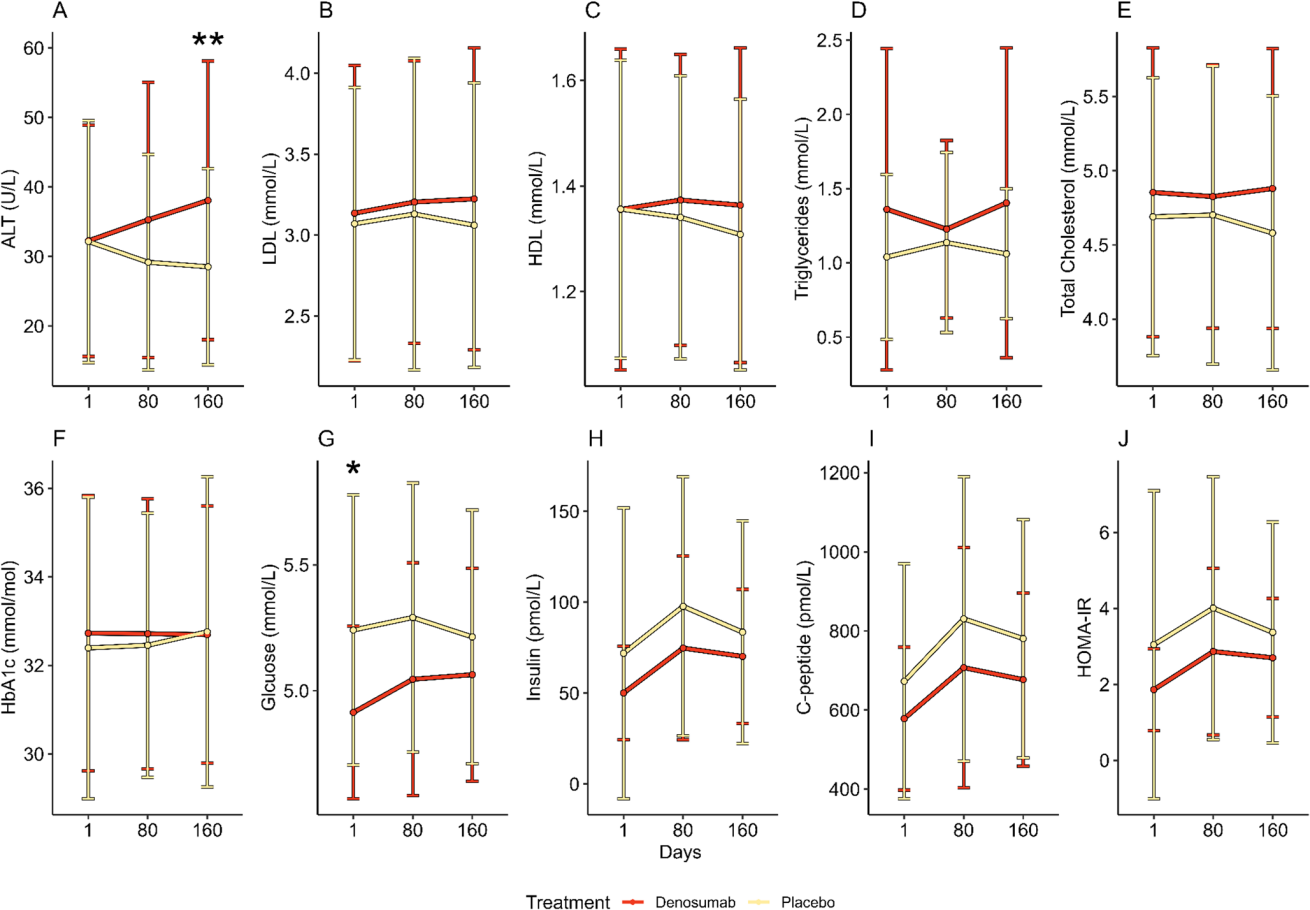
Changes in outcome presented as means (SD) for each group at day 80 and 160. **A**: Alanine aminotransferase, **B-E**: Plasma levels of LDL, HDL, triglycerides and total cholesterol. **F**: HbA1c **G-J**: Values only presented for fasting patients (*n* = 22 in each group), plasma levels of glucose, insulin, C-peptide and HOMA-IR. *P-value < 0.05, **P-value < 0.01. Abbreviations: ALT, alanine aminotransferase; HbA1c, glycated hemoglobin A1c; HDL, high-density lipoprotein; HOMA-IR, homeostatic model assessment of insulin resistance; LDL, low-density lipoprotein

In the unadjusted analysis of triglycerides, there was an increase in the denosumab group compared to placebo using bootstrapped CIs 0.3 (0.1 to 0.8). However, the Mann-Whitney-U test failed to reach significance (*p* = 0.13), and the adjusted analysis did not show any difference (Table 2). There were no differences between outcomes of HDL, LDL, or total cholesterol in the unadjusted or adjusted analysis. Trends over time are displayed in Fig. 2.

For the 44 fasting patients, FPG at baseline was significantly higher in the placebo group (data not shown). No significant effects on FPG, fasting plasma insulin (FPI), C-peptide, or HOMA-IR were observed with denosumab treatment (Table 2). A similar trend was observed in both groups with increases in all measures at day 80 and then a small decrease at day 160 relative to day 80, except for FPG in the denosumab which continued to rise at day 160 (Fig. 2).

There were no differences in lean or fat mass between the two groups at day 160 (Table 2). With denosumab both total fat and lean mass tended to increase, but the trend for lean mass reversed in the adjusted analysis.

### Subgroup Analysis

**Fig. 3:**
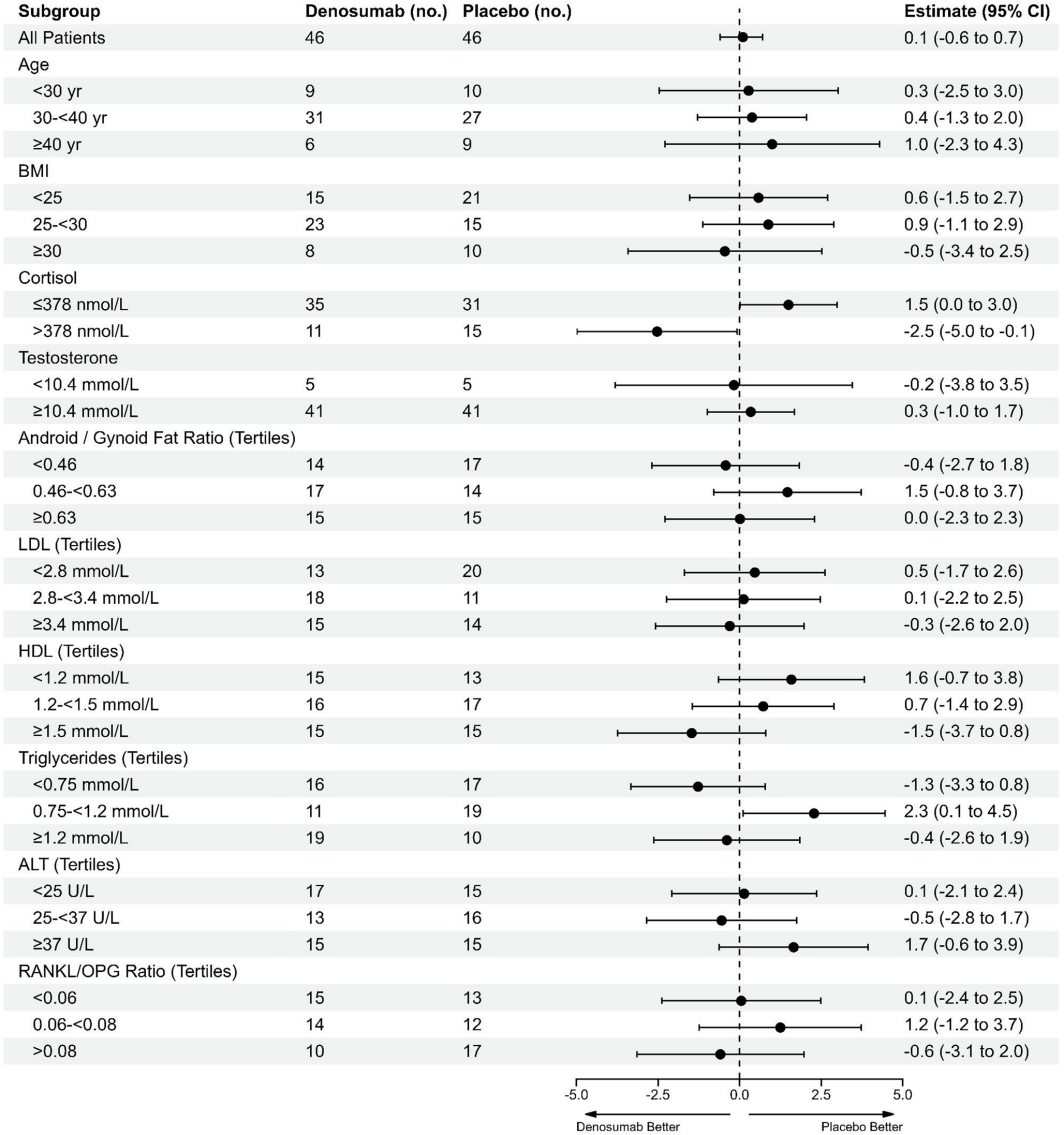
Exploratory subgroup analysis at day 80. Shown are forest plots for differences in HbA1c (mmol/mol) between treatments according to subgroups. Differences were calculated using estimated marginal means from a linear mixed model with an interaction between treatment, time and subgroup

Multiple subgroup analyses were conducted to explore the presence of any heterogeneity of treatment effect. HbA1c was chosen as the outcome for the subgroup analyses, as fasting is not a prerequisite for accurate measurement. All initial subgroups were stratified into 3 groups according to baseline variables, where an interaction was anticipated. Age was split arbitrarily at 20 and 30 and the cutoffs for BMI was 25 and 30 as used clinically. Lipids, Android/Gynoid fat ratio, sRANKL/OPG-ratio, and ALT were divided into tertiles. Cortisol as a subgroup biomarker was identified, and the cut-off at 378 nmol/L chosen by the SIDES algorithm [35]. The cutoff of 10.4 nmol/L for total testosterone was selected to align with clinical practice and the biochemical criteria for hypogonadism. All contrast analyses for the subgroups are shown in Fig. 3.

There appeared to be a significant effect in the mid tertile of the triglyceride subgroup with an increase in HbA1c, however the test for interaction was not significant (*p* = 0.23) and the difference was also seen at baseline and day 160 (data not shown). Of all the subgroups the only significant test for interaction was for the cortisol subgroup (*p* = 0.0006). The interaction was still significant after modelling cortisol as a continuous variable (*p* = 0.005). There was a reduction in HbA1c of -2.5 mmol/mol (-5.0 to -0.1) (*p* = 0.044) in the high cortisol group and an increase of 1.5 mmol/mol (0.0 to 3.0) (*p* = 0.048) in the low cortisol group. There were no significant subgroup interactions in any subgroup at day 160 (data not shown). There was no difference between FPG, HOMA-IR or fasting insulin in the cortisol subgroup (data not shown).

## Discussion

This study shows that denosumab has no major impact on glucose and lipid homeostasis in a cohort of young infertile men. The RANKL/RANK/OPG signaling axis has been implicated in hepatic inflammation, insulin sensitivity, and β-cell function [13, 16–18], but in this study, a single denosumab injection had no significant effect on serum HbA1c. Only the liver/muscle enzyme ALT was increased by denosumab in this cohort. Pre-clinical studies suggest that RANKL inhibition improves insulin sensitivity only in situations with increased RANKL expression or a high-fat diet (HFD) [10, 15, 18]. This requirement may explain the absence of glycemic effects in this cohort. These findings are in accordance with a systematic review, which reported no significant effect of denosumab in NGT individuals [30]. While infertile men likely represent a more metabolically unhealthy population group than fertile men [2, 3], denosumab still failed to exert its glycemic effects in this cohort. The same is evident for fasting- glucose, insulin, C-peptide, and HOMA-IR, as denosumab administration did not significantly affect any of these variables. Similar trends were observed in both treatment groups over time in these variables, with a modest increase at day 80 and a smaller decrease at day 160. These trends may reflect the concurrent administration of vitamin D, which has been shown to modulate beta-cell function, insulin receptor activity, and inflammatory cytokine production in pre-clinical studies [36]. These trends contrast with prior observations by our group, where vitamin D and calcium supplementation improved fasting insulin in infertile men [37]. However, the included men in the previous cohort were all vitamin D deficient and treated with significantly higher doses. Taken together, the present data confirm that denosumab exerts no effects on glucose metabolism in a normoglycemic cohort. Beyond glucose metabolism, denosumab demonstrated no detectable impact on lipid profiles. Although RANKL and OPG are expressed in adipocytes, the function of the RANKL/RANK/OPG axis in lipid metabolism remains undefined [38]. Consistent with prior clinical trials, denosumab treatment did not alter serum lipid concentrations [22–24]. Reports of hypercholesterolemia as a side effect in regulatory documents likely reflect differences in statin initiation during the FREEDOM trial. Furthermore, no significant changes in lean or fat mass were observed. Although denosumab prevented increases in android and gynoid fat mass in premenopausal breast cancer patients, these patients underwent estradiol suppression therapy, and the effects only reached significance after 12 months [24]. Collectively, these results indicate that a single injection of denosumab has minimal effects on lipid metabolism and body composition.

Denosumab administration resulted in a small, significant increase in ALT levels by day 160. Although metabolic dysfunction-associated steatotic liver disease (MASLD) remains the primary cause of elevated ALT, OPG-mediated hepatic lipid accumulation appears unlikely, as denosumab has not been shown to activate this pathway. Previous trials have not reported ALT elevations following denosumab use, and it is labeled as non-hepatotoxic [39]. Skeletal muscle, which expresses more RANKL than liver tissue [15], may instead represent the source of increased ALT, since young men on average have substantially more lean mass than post-menopausal women, the primary population in which denosumab has been studied. However, no changes in lean body mass were detected. Therefore, these findings suggest that ALT elevations are minor and unlikely to reflect clinically relevant hepatic or muscular injury.

In a small subgroup of participants with baseline plasma cortisol levels exceeding 378 nmol/L, a 2.5 mmol/mol reduction in HbA1c was observed, but no difference was seen in FPG, FPI, C-peptide, or HOMA-IR. Although elevated cortisol may upregulate RANKL expression [40], no interaction with sRANKL was detected. The absence of changes in FPG suggests impaired glucose tolerance rather than impaired fasting glucose and thus predominant peripheral- rather than hepatic insulin resistance. This subgroup could reflect a different phenotype of IGT patients, consistent with denosumab only showing glycemic effect in this population [30].

A major limitation of this study was that more than half of the participants did not do an overnight fast as instructed before blood sampling. This severely reduced the power to detect changes in indices of insulin resistance. Furthermore, stratification by fasting status before and after randomization reintroduces the potential for confounding, as treatment with denosumab could influence fasting status due to gastrointestinal side effects or upper respiratory tract infection. Subgroup analyses were not pre-specified or adjusted for multiple testing and should be interpreted with caution and purely as hypothesis-generating. The study is exploratory rather than confirmatory due to the secondary and non-pre-specified nature of the outcomes. Still, this study represents the largest randomized trial to examine denosumab’s effect on HbA1c in normoglycemic young males. The narrow confidence intervals around HbA1c in the adjusted analysis suggest that the study had adequate power to exclude a clinically significant difference in this cohort. However, evidence of no effect should be interpreted with caution due to the exploratory nature.

These findings support the notion that denosumab only exerts measurable glycemic effects if an underlying metabolic dysregulation, such as impaired glucose tolerance, is present. The observed interaction with cortisol identifies a possible treatment-sensitive subgroup that warrants further investigation.

## Conclusion

In a cohort of young infertile men, inhibition of RANKL with a single dose of denosumab showed no evidence of major effects on glucose- or lipid homeostasis, while the post hoc effect in men with highest serum cortisol warrants verification in larger studies.

## Supplementary Information

Below is the link to the electronic supplementary material.

Supplementary Material 1

## Data Availability

Some or all datasets generated during and/or analyzed during the current study are not publicly available but are available from the corresponding author on reasonable request.
