# Supplementary material for: Effects of a single-dose denosumab on glucose and lipid homeostasis in young infertile men
Source: Endocrine. 2026 Jun 25;91(1):215. doi: 10.1007/s12020-026-04683-8 (PMC13303462; doi:10.1007/s12020-026-04683-8)
Supplement: Supplementary file 1 — Supplementary Material 1 [file 12020_2026_4683_MOESM1_ESM.pdf]

Supplementary Data

Baseline Table (mean (SD)) for Fasting Patients	Denosumab	Placebo
**Included**	22	22
**Age**	34.3 (7.2)	32.2 (5.2)
**Height (cm)**	182.4 (6.9)	184.3 (9)
**Weight (kg)**	89.1 (14.2)	87.2 (12.9)
**BMI**	26.8 (3.7)	25.6 (3)
**Smokers**		
- **Active**	5 (22.7%)	3 (13.6%)
- **Never**	11 (50.0%)	13 (59.1%)
- **Previous**	6 (27.3%)	6 (27.3%)
**Glucose (mmol/L)**	5.2 (0.5)	4.9 (0.3)
**Insulin (pmol/L)**	71.9 (80)	50.1 (25.8)
**C-peptide (pmol/L)**	672.7 (297)	578.3 (180.7)
**HbA1c (mmol/mol)**	33.5 (3.3)	32.2 (2.7)
**HOMA-IR**	3 (4.1)	1.9 (1.1)
**Total Cholesterol (mmol/L)**	4.8 (1)	4.7 (0.9)

**HDL (mmol/L)**	1.4 (0.3)	1.4 (0.3)
**LDL (mmol/L)**	3.2 (1)	3 (0.9)
**Triglycerides (mmol/L)**	1.2 (0.7)	1 (0.5)
**ALT (U/L)**	32.8 (16.9)	28.5 (11.4)
**Cortisol nmol/L**	323.8 (115.1)	305.2 (64.8)
**Testosterone (nmol/L)**	18.4 (4.6)	19.5 (7.9)
**Hypogonadism (%)**	0 (0)	3 (13.6)
**Total Fat Mass (kg)**	24.7 (9.1)	22.5 (7.3)
**Android Fat Mass (kg)**	2.4 (1.3)	2.0 (0.9)
**Gyneoid Fat Mass (kg)**	3.9 (1.4)	3.8 (1.3)
**Total Lean Mass (kg)**	60.3 (7.1)	60.6 (7.5)
**Android Lean Mass (kg)**	4.1 (0.5)	4.0 (0.5)
**Gyneoid Lean Mass (kg)**	9.7 (1.3)	9.9 (1.3)

Supplementary Table 1:

Baseline characteristics for fasting participants. Data presented as means and SD or frequencies and percentages. Abbreviations: ALT, alanineaminotransferase; BMI, body mass index; HbA1c, glycated hemoglobin A1c; HDL, high-density lipoprotein; HOMA-IR, homeostatic model assessment of insulin resistance; LDL, low-density lipoprotein; HDL, high-density lipoprotein. Hypogondism defined by serum testosterone < 10.4 nmol/l.
